# A Machine-Learning Method of Predicting Vital Capacity Plateau Value for Ventilatory Pump Failure Based on Data Mining

**DOI:** 10.3390/healthcare9101306

**Published:** 2021-09-30

**Authors:** Wenbing Chang, Xinpeng Ji, Liping Wang, Houxiang Liu, Yue Zhang, Bang Chen, Shenghan Zhou

**Affiliations:** 1 School of Reliability and Systems Engineering, Beihang University, Beijing 100191, China; changwenbing@buaa.edu.cn (W.C.); sy1914103@buaa.edu.cn (X.J.); zy1914125@buaa.edu.cn (H.L.); Zhangyue1127@buaa.edu.cn (Y.Z.); bang@buaa.edu.cn (B.C.); 2 Department of Neurology, Peking University Third Hospital, Beijing 100191, China; abn8360@gmail.com

**Keywords:** ventilatory pump failure, vital capacity plateau value, biomedical engineering, RFECV, disease prediction, LightGBM

## Abstract

Ventilatory pump failure is a common cause of death for patients with neuromuscular diseases. The vital capacity plateau value (VCPLAT) is an important indicator to judge the status of ventilatory pump failure for patients with congenital myopathy, Duchenne muscular dystrophy and spinal muscular atrophy. Due to the complex relationship between VCPLAT and the patient’s own condition, it is difficult to predict the VCPLAT for pediatric disease from a medical perspective. We established a VCPLAT prediction model based on data mining and machine learning. We first performed the correlation analysis and recursive feature elimination with cross-validation (RFECV) to provide high-quality feature combinations. Based on this, the Light Gradient Boosting Machine (LightGBM) algorithm was to establish a prediction model with powerful performance. Finally, we verified the validity and superiority of the proposed method via comparison with other prediction models in similar works. After 10-fold cross-validation, the proposed prediction method had the best performance and its explained variance score (EVS), mean absolute error (MAE), mean squared error (MSE), root mean square error (RMSE), median absolute error (MedAE) and R^2^ were 0.949, 0.028, 0.002, 0.045, 0.015 and 0.948, respectively. It also performed well on test datasets. Therefore, it can accurately and effectively predict the VCPLAT, thereby determining the severity of the condition to provide auxiliary decision-making for doctors in clinical diagnosis and treatment.

## 1. Introduction

The central nervous system, peripheral nervous system, neuromuscular tissue and thorax that drive or regulate respiratory movement are collectively referred to as the ventilatory pump. Respiratory failure caused by dysfunction in these parts is called ventilatory pump failure. Pump failure mainly causes patients to have ventilatory dysfunction, manifested as type II respiratory failure. Common neurological diseases causing ventilatory pump failure include brain trauma, brain stroke, brain tumor, encephalitis, myelitis, motor neuron disease, acute inflammatory polyradiculoneuropathy, myasthenia gravis, polymyositis, muscular dystrophy and drug poisoning.

Ventilatory pump failure can be an acute on chronic condition that without ventilatory assistance leads to poor prognosis and even death in patients with critical neurological diseases. The rapid diagnosis and accurate treatment of ventilatory pump failure can effectively reduce the mortality of patients and provide opportunities for the recovery of nervous system function. Therefore, it is important to determine the disease severity for pediatric diseases to prevent sudden deterioration of the condition. In order to do this, it is helpful to monitor changes in the relevant medical indicators. CA Pandit et al. described the current evidence for daytime pulmonary function tests and their ability to predict imminent respiratory morbidity [1].

For children with certain diseases (such as Duchenne muscular dystrophy), the vital capacity plateau value (VCPLAT) is one of the key indicators. VCPLAT refers to the maximum value that the lung capacity of pediatric patients with neuromuscular disease (normally congenital myopathy, Duchenne muscular dystrophy and spinal muscular atrophy) can rise to and remain unchanged for a period of time. Once vital capacity drops from this value, it indicates that the condition has deteriorated, leading to aggravation of ventilatory pump dysfunction. The vital capacity plateaus on normal people at 19–20 years of age. In Duchenne muscular dystrophy (DMD) the VCPLAT at mean age 13 (range 9–17) depending on how rapidly progressive the dystrophy. In spinal muscular atrophy (SMA) type 1 the vital capacity (VC) can be highest at birth then go to zero or as late as age 4 or later because SMA is not rapidly progressive.

Predicting the patient’s VCPLAT in advance can help doctors take preventive measures such has indicating when to introduce lung volume recruitment to possibly preserve VC, thereby reducing the severity of respiratory pump dysfunction. For example, Rideau Y Jack pointed out that for respiratory function in the muscular dystrophies, the vital capacity (VC) underwent ascending, plateau and descending stages during the course of the disease. The VC at the plateau stage (VCPLAT) may be used as an estimate of life span. The severity of DMD, including the prognosis of severe scoliosis and the need for ventilatory support, have been confirmed as a function of VCPLAT [2]. Bach JR describes and correlates VCPLAT with spinal muscular atrophy (SMA) severity and prognosis for autonomous breathing. The results indicate that vital capacity should be monitored from birth. The VCPLAT correlates with prognosis with SMA 1A VCs not exceeding 100 mL or 1B 200 mL. Patients who attained 200 mL at any time (milder 1C) retain some ability to breathe after 10 years of age [3]. In order to consider the effect of active lung volume recruitment on rate of decline in VC, M Chiou introduced air stacking using volume-preset ventilation or manual resuscitator bag to all patients after they reached their VCPLAT with a lifetime maximum. The results show that for patients with DMD, active lung volume recruitment may help to preserve vital capacity and effects on post-plateau vital capacity may be a useful outcome measure for therapeutic trials [4]. Existing research showed that medical practitioners have studied the relationship between VCPLAT and disease severity, and some follow-up treatment methods based on VCPLAT have been established. If the patient’s VCPLAT can be known in advance, the delay of treatment can be avoided and the doctor can take corresponding measures in advance. Therefore, the prediction of VCPLAT is an important medical problem. However, due to the complex relationship between VCPLAT and the patient’s own condition, it is relatively difficult to predict VCPLAT from a medical perspective.

Machine learning has achieved many successful applications in the medical field [5,6,7,8,9,10,11]. This provides new ideas for VCPLAT prediction. The existing relating research involving machine learning mainly focus on the diagnosis and classification of neuromuscular diseases based on electromyography (EMG) signals and pay less attention to pump failure prediction [12,13,14,15,16,17,18,19]. For example, A Subasi’s showed that PSO-SVM can diagnose neuromuscular diseases, and the overall accuracy on 1200 EMG signals was 97.41% [15]. A Subasi also uses different machine learning models to automatically classify EMG signals as normal, neurogenic or myopathy [16]. NF Güler et al. used support vector machines (SVM) and artificial neural network (ANN) to classify EMG signals of normal, neuropathy and myopathy subjects. The best percentage of correct classification is obtained in SVM with 94.4%. In ANN, the correct classification value is 91.6%. SVM shows a higher performance with AUC value 0.954 while it is 0.927 in ANN [17]. SB Choi et al. used four different feature selection methods to achieve the efficient and rapid classification of 11 neuromuscular diseases. The study found that when the classification models of SVM-one-versus-one (OVO), SVM-one-versus-rest (OVR) and directed acyclic graph SVM (DAGSVM) are used together with the ratio of genes between-categories to within-category sums of squares (BW) feature selection method, it can distinguish neuromuscular diseases from the control group with 100% accuracy using only four features [19].

By analyzing the medical data of neuromuscular patients from a large American center, this study aimed to complete three tasks. First, we used correlation analysis and RFECV to automatically select high-quality feature combinations to identify key factors of VCPLAT among a large number of medical indicators. Second, we proposed a new method based on LightGBM to predict the VCPLAT of patients with neuromuscular diseases. Third, we evaluated the predictive performance of the model and compared it with other common machine learning models in similar works to prove the advantages of the proposed method.

This article is divided into five parts. The specific organizational structure is as follows: Section 1 mainly introduces the research background, literature review, research contents and ideas. Section 2 introduces the materials and methods and proposes a VCPLAT prediction model based on RFECV and LightGBM. Section 3 is the practical application of the proposed model, the results of which are analyzed and discussed to verify the effectiveness of the proposed method. Section 4 is the discussion. Section 5 is the conclusion.

## 2. Materials and Methods

### 2.1. Data Description

**Figure 1:**
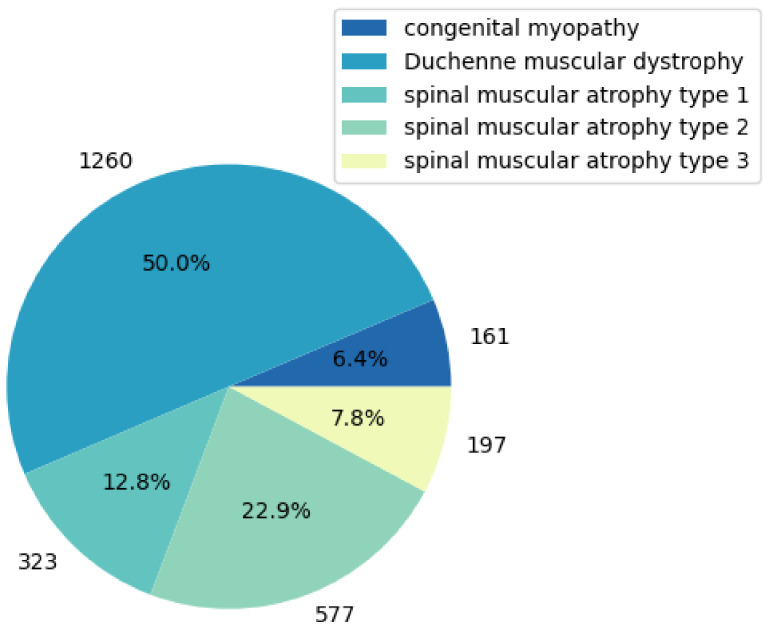
Types of diseases involved in the data and distribution of records.

The research data came from a neuromuscular disease clinic in the United States, provided by Professor Bach from Rutgers New Jersey Medical School, who collected and compiled the electronic medical record data of 3103 neuromuscular disease patients over the past 30 years. According to the purpose of the study, we selected the part of the data where the VCPLAT was recorded which is only for pediatric diseases (Duchenne muscular dystrophy, congenital myopathy and spinal muscular atrophy type 1–3). The data used in this study totaled 2518 medical records of 704 patients, involving 40 features and 5 pediatric diseases. Table 1 shows the name, description, mean, std, data type, data distribution interval and other data information of some medical indicators in the data set. In Appendix A, Table A1 shows the all 40 features and their explanations. Figure 1 shows the types of diseases involved. Due to the large number of missing values, outliers and duplicate values in the data, this study first preprocessed the data.

### 2.2. Data Preprocessing

#### 2.2.1. Missing Value Processing by Deletion and KNN

The proportion of missing data in some samples or features is relatively large. They are meaningless for research because they contained little useful information [20]. So, this study chooses to delete them directly. The deletion rules are as follows:

(1) Delete features with missing values exceeding 50%.
(2) Delete samples with missing values exceeding 50%.

For the parts that still have missing values afterwards, this study uses the KNN (k-Nearest Neighbor) algorithm for interpolation, a good and normal way of handling missing value [21]. K represents the number of samples closest to the sample to be filled and it is a user-defined constant [22]. The idea of this algorithm is to find K similar samples for a sample with missing values and fill in the missing parts according to the characteristics of similar samples. There is no specific method, in determining the K value for the KNN missing data imputation [23,24]. For example, U Pujianto et al. used the KNN method as an imputation carried out in several cases with different mechanisms and missing data model, and set k to 1, 3, 5 and 7, respectively. The results show that handling missing data with KNN-based imputation can reach the accuracy of complete data in each case with a low accuracy difference [23]. If K is too large, it is easy to cover samples with farther similarity. If K is too small, the filling value is easily affected by noise samples. In practical applications, it is generally recommended to use a relatively small K value [25]. In this study, K was determined to be 3. The filling rules are as follows:

(1) If the missing variable to be filled is a discrete variable, the K nearest neighbor classifier is used to fill in the missing part with the most frequent category of the K neighbors.
(2) If the missing variable to be filled is a continuous variable, the K nearest neighbor regressor is used to fill in the missing part with the average value of the variable among K neighbors.

#### 2.2.2. Category Feature Conversion

Generally, in the medical field, the database may involve all the type of small or extensive categorical dataset, which makes the disease prediction analysis more complicated [26]. Most machine learning algorithms require the input to be numeric data; therefore, the category features need to be converted. The category with no inherent order is called Nominal Feature, such as gender (male/female), which can be converted in the form of one-hot coding. The category with an internal order is called Ordinal Feature, such as mild, normal, severe and critically ill. Due to the degree of progressive relationship, numbers can be used for direct conversion [25]. For example, L Cui et al. used the 2-dimensional one-hot vector to express the gender of a patient and used embedding methods to map each medical code to a low-dimensional continuous vector [27]. The Nominal category feature in this study is gender. The conversion standard is: male is 0, female is 1. The Ordinal category features in this study are muscle strength test indicators.

Muscle strength refers to the strength of muscle contraction when the limb is exercising freely. The strength test indicators involved in this study include: SHOULDERL, SHOULDERR, BICEPSL, BICEPSR, WRISTL, WRISTR, MPHIPFLLT, MPHIPFLRT, MPQUADLT, MPQUADRT, MPDORSILT and MPDORSIRT, a total of 12 features. The conversion criteria for this category are shown in Table 2.

#### 2.2.3. Data Normalization

As the dimensions of various medical indicators are not uniform, this study used maximum and minimum standardization to normalize the data. It is a linear transformation of the original data, which can map the data values to the [0, 1] interval. The transformation function is as follows:

(1) $$x^{*}=\frac{x-min}{max-min},$$

where *max* is the maximum value of the data, and *min* is the minimum value of the data.

### 2.3. Correlation Analysis and Significance Test

There are autocorrelation features in the data, resulting in feature redundancy. Furthermore, not all medical features are related to the vital capacity plateau. Correlation analysis aims to study the correlation between random variables [28]. For example, IY Jung et al. conducted the correlation analysis of functional factors and age with Duchenne Muscular Dystrophy. The results showed that the Brooke scale, Vignos scale and passive range of motion (PROM) of ankle dorsi-flexion were partially available to assess DMD patients [29]. Correlation coefficients that are commonly used include the Pearson correlation coefficient and Spearman’s rank correlation coefficient [28]. The former applies to data that meets a normal distribution, while the latter applies to data of any distribution. After the normality test, most of the medical data used in this study did not meet the normal distribution. Therefore, Spearman’s rank correlation coefficient was to measure the correlation among various medical indicators, which can simplify the medical feature dimensions, and preliminarily analyze the medical variables that are correlated with the VCPLAT.

The rank of a number is the average descending position of the number in the overall data. If X and Y are the two observed variables, and the sample size is *n*, then for each sample *X_i_*, *Y_i_*, the corresponding rank is *x_i_*, *y_i_*. Then, the Spearman’s rank correlation coefficient between these two variables is:

(2) $$\rho =1-\frac{6\sum d_{i}^{2}}{n\left(n^{2}-1\right)}$$

where *d_i_* = *x_i_* − *y_i_* and this represents the rank difference between the two indicators of the *i*-th sample.

The Spearman’s rank correlation coefficient ranges from −1 to 1. When the absolute value is closer to 1, this indicates that the two variables are more correlated. When the value is 1, this represents a completely positive correlation; when the value is −1, this represents a completely negative correlation; and when the value is 0, this indicates it is completely uncorrelated.

In order to confirm the authenticity of the correlation between the two variables, the significance test of the correlation coefficient should also be performed. According to the significance coefficient *p* value, the significance test standard is as follows:

*p* < 0.05: Statistical differences exist, reject the null hypothesis and the two variables are correlated.

*p* > 0.05: No statistical difference exists, accept the null hypothesis and the two variables are not correlated.

### 2.4. Recursive Feature Elimination with Cross-Validation (RFECV)

The filtered data after correlation analysis may still have a high feature dimension with a great deal of redundant information and noise, which lead to greater learning burden and lower running efficiency on the model. Therefore, feature selection is needed.

Recursive feature elimination with cross-validation (RFECV) is a wrapper feature selection. Wrapped feature selection uses the performance evaluation of the machine learning model as the evaluation criterion of the selected feature subset [30]. It selects features by recursively reducing the size of the feature set. The main steps are as designed as follows:

(1) We determine LightGBM as the base prediction learner.
(2) Use all original features as initial feature subset.
(3) Train the prediction model on the feature set. Assign a weight to each feature based on the impact of the feature on the predictor’s performance.
(4) The feature with the smallest absolute weight is kicked out of the feature set.
(5) Repeat (3) and (4) recursively until the number of remaining features reaches the best performance score.

RFECV performs RFE through cross-validation to select the optimal number of features. For a set of *n* features, the number of all its subsets is $2^{n}-1$ (excluding the empty set). RFECV calculates the validation error of all subsets through the base learner and then selects the subset with the smallest error as the final feature set.

RFECV has been successfully applied in the medical field, especially in the evaluation of the importance of medical features and the selection of medical indicators [31,32,33]. For example, K Akyol et al. showed through experiments that medical decision support systems with more accurate predictions could be developed using RFECV and this can be helpful in selecting the treatment method for the experts in the field [33].

For RFECV in this paper, the cross-validation method was a 10-fold cross-validation, the evaluation criteria were root mean square error (RMSE), mean squared error (MSE), mean absolute error (MAE), median absolute error (MedAE), explained variance score (EVS) and R^2^ Score.

### 2.5. LightGBM

LightGBM is a boosting ensemble model [34]. We train the input data (patient’s various medical indicators after RFECV) and the output data (the patient’s VCPLAT) with LightGBM to establish an integrated decision tree structure to reflect the mapping relationship between medical indicators and VCPLAT. After the training is completed, the model can predict the VCPLAT of a new patient when his medical characteristics are input. In this study, the parameter adjustment method was Grid Search.

Boosting algorithms, such as XGBoost and GBDT are powerful algorithms in the current field of machine learning [35]. For example, P Bahad et al. evaluated the use of Gradient Boosting ensemble machine learning techniques for two-class prediction. The experimental result shows that prediction accuracy of Gradient Boosting algorithm is better than traditional machine learning to classify patients with diabetes using diabetes risk factors [36]. C Chen et al. proposed a new protein interaction prediction method based on LightGBM. The results of the 5-fold cross-validation show that the accuracy of this method is significantly better than traditional prediction methods [37]. Wang D et al. compared the performance of different machine learning methods on the recognition of miRNA in patients with breast cancer, and found that LightGBM performs better in many aspects [38]. Y Amirgaliyev et al. compared and discussed four supervised machine learning algorithms on five gastric diseases detection. The results show that LightGBM has a higher accuracy on the test data set, reaching more than 95% [39]. In summary, LightGBM has mature applications in various fields of medicine, and can analyze various types of medical data to accurately classify and predict diseases.

Traditional boosting algorithms often perform poorly when dealing with large medical samples and high-dimensional data. This is because they need to scan all the sample points for each feature to select the best split point when calculating the information gain, which is obviously very time-consuming, resulting in low efficiency and scalability of the model.

LightGBM solves this problem well. Similar to GBDT and XGBoost, LightGBM uses the negative gradient of the loss function as the residual approximation of the current decision tree to fit the new decision tree. LightGBM mainly includes the following three algorithms to improve the defects of the original boosting algorithm.

The first algorithm is gradient-based one-side sampling (GOSS). Samples with large gradients have a greater impact on information gain. Therefore, GOSS retains all samples with large gradients and uses random sampling on samples with small gradients. In order to offset the impact on the data distribution, GOSS introduces a constant multiplier to the data with small gradients when calculating the information gain. Specifically, the data are first sorted in descending order according to the absolute value of their gradient and the top *a* × 100% samples are selected as data subset *A*. Then, it randomly selects *b* × 100% samples in the remaining data *A^c^* as data subset *B*. Finally, it uses the following formula to calculate the information gain of splitting feature *j* at point *d*:

(3) $${\tilde{V}}_{j}\left(d\right)=\frac{1}{n}\left(\frac{{\left(\sum_{x_{i}\in A_{l}}g_{i}+\frac{1-a}{b}\sum_{x_{i}\in B_{l}}g_{i}\right)}^{2}}{n_{l}^{j}\left(d\right)}+\frac{{\left(\sum_{x_{i}\in A_{r}}g_{i}+\frac{1-a}{b}\sum_{x_{i}\in B_{r}}g_{i}\right)}^{2}}{n_{r}^{j}\left(d\right)}\right)$$

where $A_{l}=\left\{x_{i}\in A:x_{ij}\leq d\right\}$, $A_{r}=\left\{x_{i}\in A:x_{ij}>d\right\}$, $B_{l}=\left\{x_{i}\in B:x_{ij}\leq d\right\}$, $B_{r}=\left\{x_{i}\in B:x_{ij}>d\right\}$, n is total number of samples in a training set, the coefficient $\frac{1-a}{b}$ is used to normalize the sum of the gradients over B back to the size of *A^c^* and the $g_{i}\;\left(i=1\ldots n\right)$ are the negative gradients of the loss function with respect to the output of the model in each iteration of gradient boosting. For medical data in this study, there are many patients with incomplete data records with low information value and small difference, so LightGBM can prevent such samples from affecting the prediction model.

The second algorithm is exclusive feature bundling (EFB). The high-dimensional feature space is often very sparse, and there are many mutually exclusive features. Therefore, some features can be bundled into one feature, thereby reducing the dimension of the feature and the consumption of finding the best cut point. Specifically, EFB first sorts the features according to the number of non-zero values. Then, it calculates the conflict ratio between the different features. Next, it iterates over each feature and attempts to merge features to minimize the conflict ratio.

**Figure 2:**
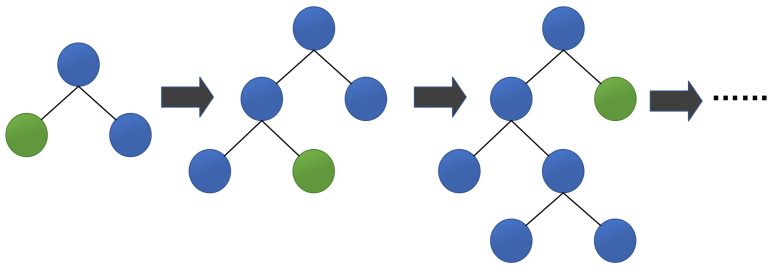
Leaf-wise tree growth strategy.

**Figure 3:**
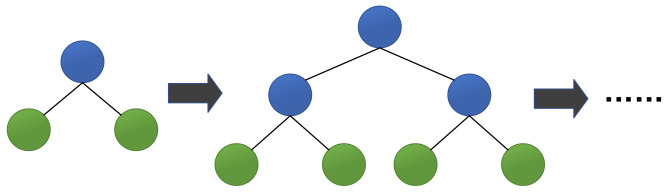
Level-wise tree growth strategy.

The third algorithm is Leaf-wise, an efficient decision tree growth strategy with depth limitation. As shown in Figure 2, LightGBM finds the leaf with the largest split gain from all the current leaves, and then splits the leaf. Then it continuously loops this process. In contrast, XGBoost uses a Level-wise growth strategy as shown in Figure 3, which can split the leaves of the same layer at the same time. Many leaves have a low split gain, so there are many unnecessary searches and splits. Therefore, compared with Level-wise, in the case of the same number of splits, Leaf-wise has a lower error and higher accuracy. The limitation of the maximum depth prevents overfitting.

In summary, LightGBM is a fast, distributed, high-performance gradient boosting framework based on the decision tree algorithm. It uses GOSS and EFB to reduce the amount of calculated data and the number of features, respectively, without losing its accuracy. Using Leaf-wise ensures faster training efficiency. LightGBM can process large-scale medical data, support parallel learning and achieve low memory usage with high accuracy.

### 2.6. Model Evaluation Methods

In order to evaluate the performance of the prediction model, we adopted the following evaluation criteria: Explained variance score (EVS), Mean absolute error (MAE), Mean squared error (MSE), Root mean squared error (RMSE), Median absolute error (MedAE) and R2 Score.

Assuming that *y_i_* is the true value of the *i*-th sample, *f_i_* is the corresponding predicted value and $\bar{y}$ is the mean of *y*, i.e., $\bar{y}=\frac{1}{n}\sum_{i=1}^{n}y_{i}$, then the evaluation indexes of n samples are given by the following formulas.

(1) Explained Variance Score (EVS):

(4) $$\mathrm{Explained}\;\mathrm{Variance}=1-\frac{\sum_{i=1}^{n}{\left(\left(y_{i}-f_{i}\right)-\bar{\left(y_{i}-f_{i}\right)}\right)}^{2}}{\sum_{i=1}^{n}{\left(y_{i}-\bar{y}\right)}^{2}}$$

where $\bar{y_{i}-f_{i}}$ represents the mean of $y_{i}-f_{i},$ i.e., $\bar{y_{i}-f_{i}}=\frac{1}{n}\sum_{i=1}^{n}(y_{i}-f_{i})\;$. EVS measures how close the difference between all predicted values and the sample is to the sample itself. The maximum value is 1. The closer to 1, the better.

(2) Mean Absolute Error (MAE):

(5) $$\mathrm{MAE}=\frac{1}{n}\overset{n}{\underset{i=1}{\sum }}\left|f_{i}-y_{i}\right|$$

MAE is used to describe the difference between the predicted value and the true value. The smaller the value, the better.

(3) Mean Squared Error (MSE):

(6) $$\mathrm{MSE}=\frac{1}{n}\overset{n}{\underset{i=1}{\sum }}{\left(f_{i}-y_{i}\right)}^{2}$$

Mean Squared Error, also known as Mean Squared Deviation (MSD), measures the degree to which the overall sample deviates from the predicted value of the model. The smaller the value, the better.

(4) Root Mean Squared Error (RMSE):

(7) $$\mathrm{RMSE}=\sqrt{\frac{1}{n}\sum_{i=1}^{n}{\left(f_{i}-y_{i}\right)}^{2}}$$

The RMSE is very sensitive to the large or small error in a group of measurements, so it can reflect the precision of the measurement well. The smaller the value, the better.

(5) Median Absolute Error (MedAE):

(8) $$\mathrm{MedAE}\left(y,f\right)=median\left(\left|y_{1}-f_{1}\right|,\ldots ,\left|y_{n}-f_{n}\right|\right)$$

The MedAE can reduce the effect of outliers. The smaller the value, the better.

(6) R2 Score:

(9) $$R^{2}=1-\frac{\sum_{i=1}^{n}{\left(y_{i}-f_{i}\right)}^{2}}{\sum_{i=1}^{n}{\left(y_{i}-\bar{y}\right)}^{2}}$$

R2 Score, also called the Coefficient of Determination, judges the degree of fit between the predicted value and the real data. The best value is 1, and it can be negative. The closer to 1, the better.

**Figure 4:**
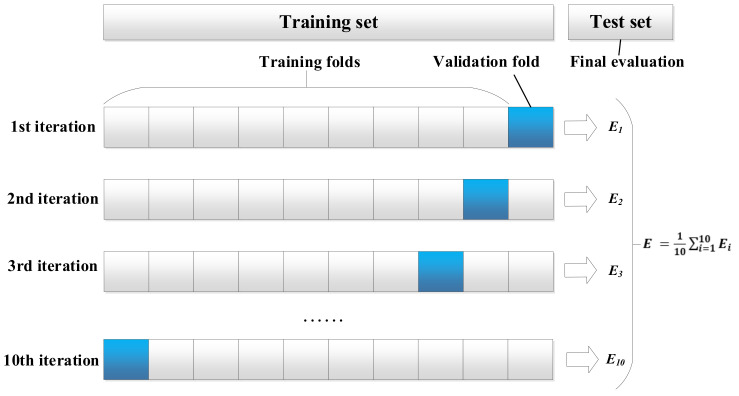
10-fold cross-validation and test set evaluation methods. *E* represent the results of the regression evaluation criteria such as EVS, MAE, MSE, RMSE, MedAE, R2.

The verification methods included 10-fold cross-validation and test set evaluation (Figure 4). We divided the medical data into training set, validation set and test set. The training set and the validation set account for 70% of the data volume and are used for 10-fold cross training and validation. The test set accounts for 30% of the data volume and is completely independent from the model optimization procedure. The evaluation results of the test set can show the generalization performance of the model. After verification, we use statistical tests (Friedman-Nemenyi test) to evaluate the significance of the results.

### 2.7. Research Process

**Figure 5:**
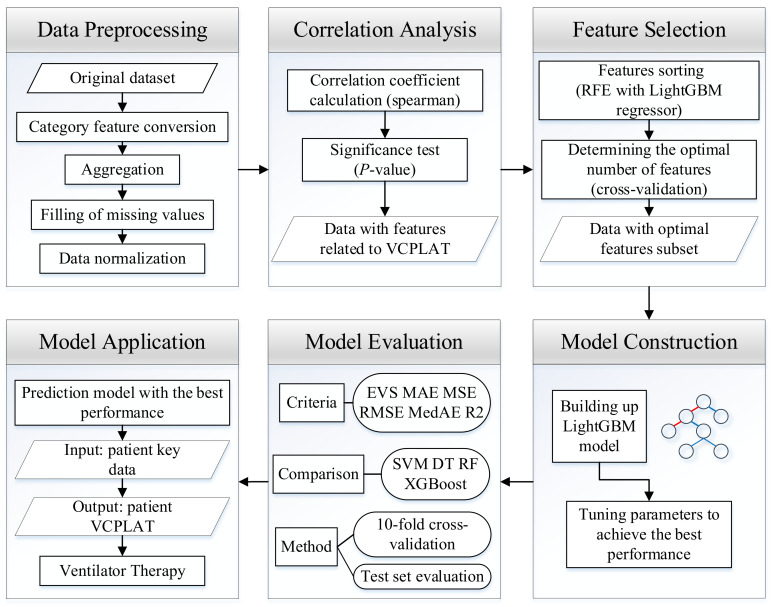
Prediction model construction process and application.

The research process is shown in Figure 5. First, the data from patients with neuromuscular disorder is obtained. In order to eliminate missing values, duplicate values, outliers, etc. in the data, this study performed data preprocessing. It quantifies text features through categorical feature conversion, deals with missing values by deletion and KNN interpolation and finally carries out data normalization to obtain a unified dimension. Then, in order to reduce the degree of data autocorrelation, and to analyze the correlation of VCPLAT, we use Spearman’s rank correlation coefficient and significance test for correlation analysis. Next, since not all features have impact on the prediction model, we use RFECV to determine the best feature subset and feature number. On this basis, we construct a prediction model of VCPLAT based on LightGBM, using the extracted data set for training and grid search method to adjust the parameters to optimize the model during the training process. Finally, we use six evaluation indicators through 10-fold cross-validation as well as test set evaluation and compares it with four machine learning models to evaluate the results. In the end, we can obtain a highly-efficient prediction model with the best performance which can output the patient’s VCPLAT when their medical indicators are input.

### 2.8. Computation Environments

All data analysis methods and algorithm implementations in this study are written by Python 3 language for programming and run on the professional version of PyCharm (Anaconda).

## 3. Results

### 3.1. Data after Preprocessing

**Figure 6:**
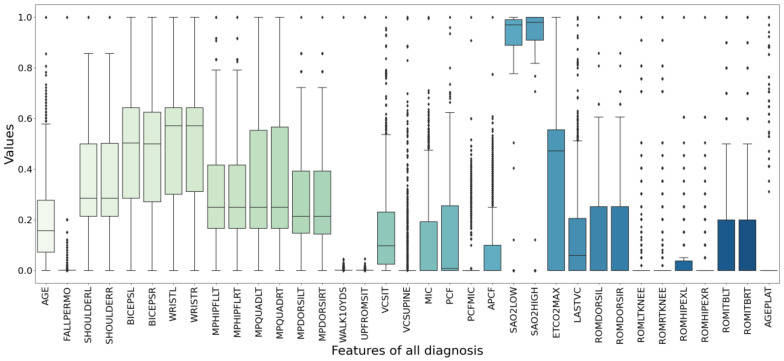
Data box plot after preprocessing. The middle horizontal line represents the median value of the data. The positions of the two ends of the rectangular box correspond to the upper and lower quartiles of the data, respectively. The two lines outside the rectangular box are the upper and lower limits of the data. Discrete points represent outliers.

**Figure 7:**
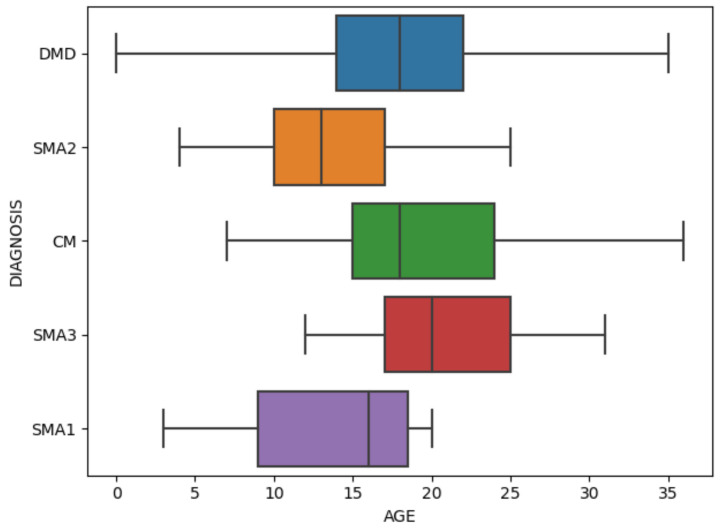
Box plot of age distribution.

**Figure 8:**
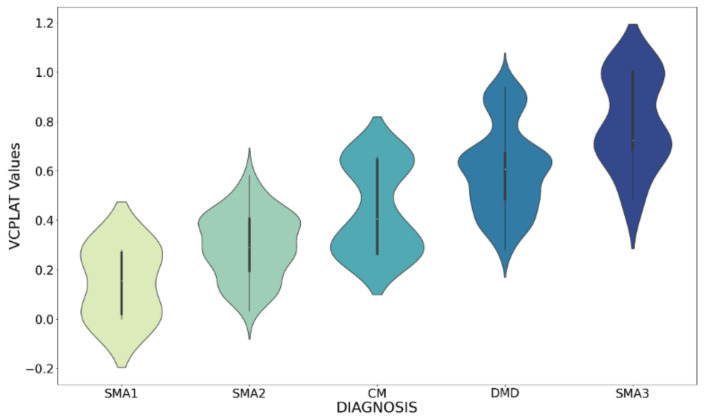
Violin plot of VCPLAT for different diagnoses.

The box plot in Figure 6 shows the data distribution after data preprocessing. It can display the maximum, minimum, median, outlier and upper and lower quartiles of a set of data. There are 494 samples and 37 features in total. Figure 7 specifically shows the age distribution of different diagnosis. After standardization, the values of all variables are between 0 and 1. Figure 8 shows the violin chart of VCPLAT distribution under different diagnoses. It displayed the distribution status and probability density of multiple sets of data.

### 3.2. Feature Correlation Coefficient Analysis Results

**Figure 9:**
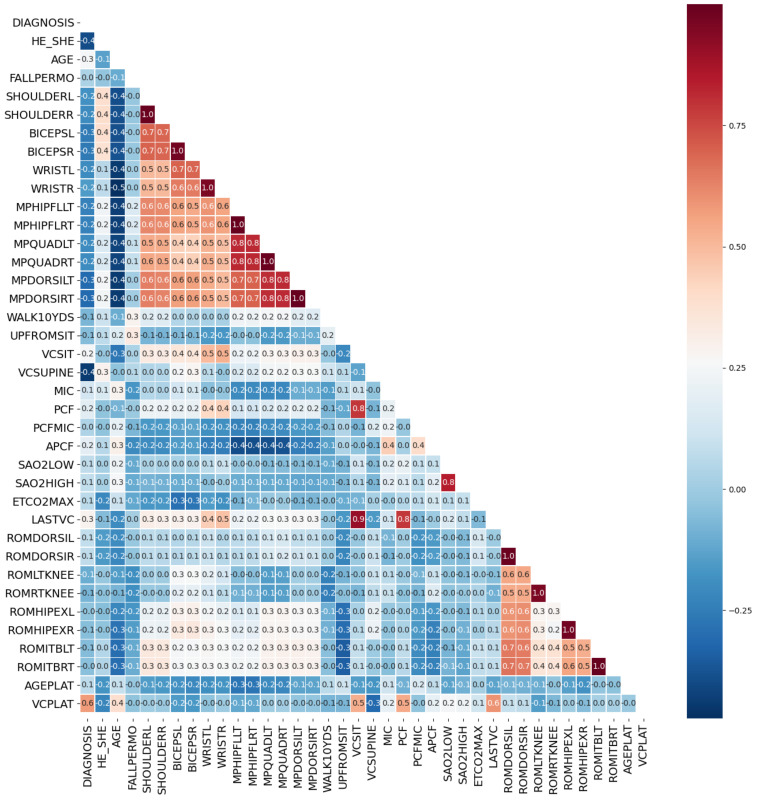
Correlation coefficient matrix heat map. The horizontal and vertical axes represent medical features, and the numbers in the small squares represent the Spearman correlation coefficients between the two features. Color represents the degree of correlation. The larger the correlation coefficient, the darker the color, which means that the two features are more related.

In this study, by calculating the Spearman’s rank correlation coefficient, the correlation between each two features was obtained, thereby the correlation coefficient matrix of medical features was constructed, as shown in Figure 9.

**Figure 10:**
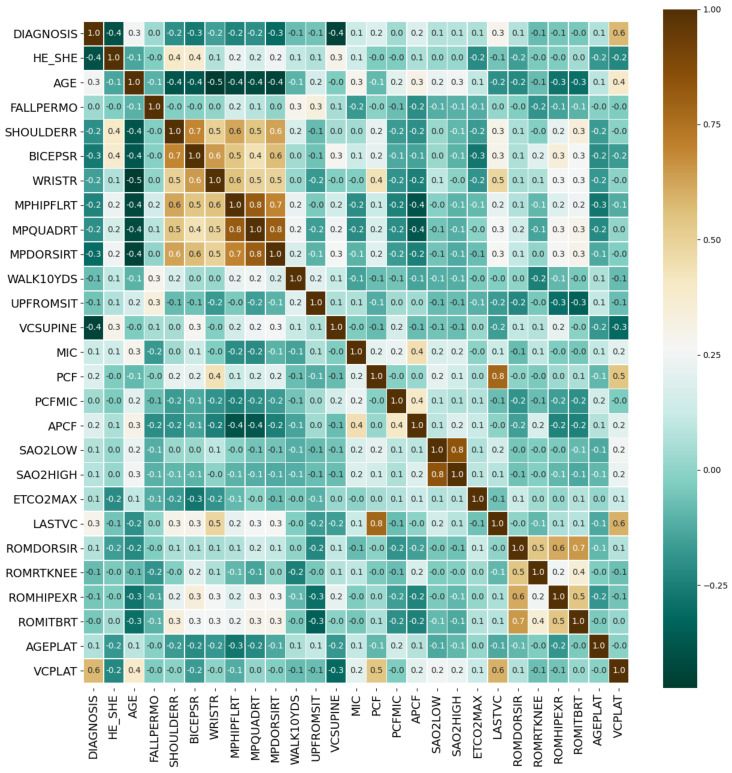
Optimized VCPLAT correlation coefficient matrix heat map. The horizontal and vertical axes represent medical features, and the numbers in the small squares represent the Spearman correlation coefficients between the two features. Color represents the degree of correlation. The larger the correlation coefficient, the darker the color, which means that the two features are more related.

The threshold for judging two variables as redundant features is that the Spearman correlation coefficient exceeds 0.95. Highly correlated feature pairs include [SHOULDERL, SHOULDERR], [BICEPSL, BICEPSR], [WRISTL, WRISTR], [MPHIPFLLT, MPHIPFLRT], [MPQUADLT, MPQUADRT], [MPDORSILT, MPDORSIRT], [ROMDORSIL, ROMDORSIR], [ROMLTKNEE, ROMRTKNEE], [ROMHIPEXL, ROMHIPEXR] and [ROMITBLT, ROMITBRT]. Indexes with a high degree of autocorrelation are basically muscle strength test indexes. Based on Figure 9, we retained only one of the two highly autocorrelated features to obtain the optimized medical feature set with simplified number of variables. The corresponding correlation matrix was shown in Figure 10.

After the significance test, features whose *p*-value was less than 0.05 included DIAGNOSIS, HE_SHE, AGE, BICEPSR, MPHIPFLRT, VCSUPINE, MIC, PCF, APCF, SAO2LOW, SAO2HIGH, ETCO2MAX and LASTVC, a total of 13 features. We rejected the original hypothesis and considered that they have a correlation with VCPLAT.

### 3.3. Feature Selection Based on RFECV

**Figure 11:**
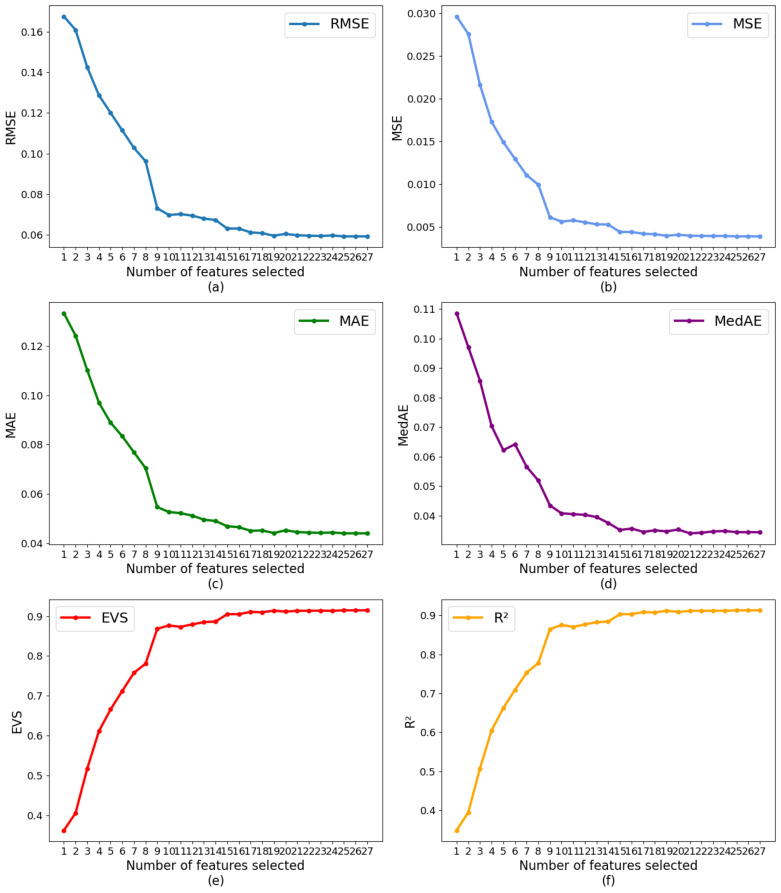
RFECV results under different evaluation criteria: (**a**) RMSE; (**b**) MSE; (**c**) MAE; (**d**) MedAE; (**e**) EVS; (**f**) R^2^.

Based on LightGBM and 10-fold cross-validation, this study used RFECV to select features from the optimized dataset by correlation analysis. The results are shown in Figure 11, which depicts the trend of the model evaluation value as the number of retained features increases under different evaluation criteria.

According to Figure 11, as the number of features increases, the performance of the model gradually tends to be flat. When the number of features is 19, there is no significant change in model performance. Taken together, the optimal number of features was 19.

**Figure 12:**
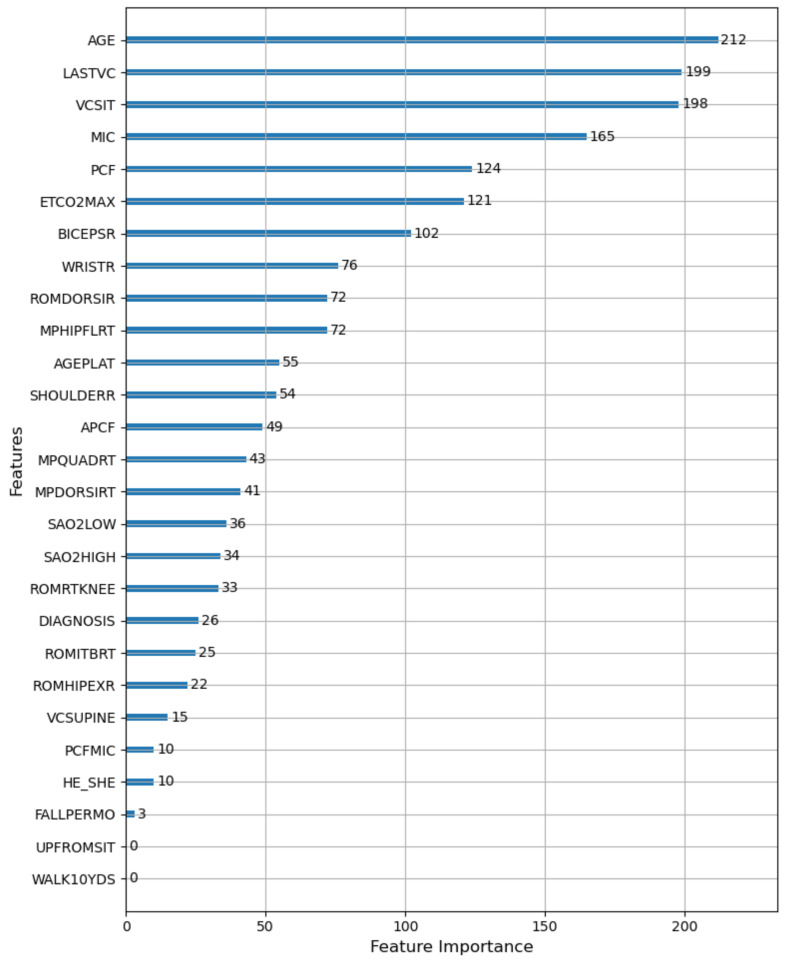
VCPLAT feature importance ranking. The numbers in the figure represent the number of times the feature is used (splited) in LightGBM.

According to the total number of times the features are used for splitting in all decision trees of LightGBM, we obtained the importance ranking of the features. The results are shown in Figure 12. According to Figure 12, age (AGE) had the greatest influence on the prediction result of VCPLAT, followed by the sitting vital capacity (VCSIT) and its value at the last visit (LASTVC). The maximum blowing capacity (MIC), peak cough flow rate (PCF), maximum end-tidal carbon dioxide (ETCO2MAX) and right bicep strength (BICEPSR) had a certain effect on VCPLAT, while the gender (HE-SHE) and fall to the ground every month (FALLPERMO) had a weak influence on VCPLAT. How fast the patient can walk within 10 yards (WALK10YDS) and time for patients to stand up from sitting on the floor (UPFROMSIT) had almost no effect on VCPLAT prediction.

In summary, we selected the first 19 features in the feature importance ranking as the best feature combination and as the input variables of LightGBM.

### 3.4. Prediction Model Based on LightGBM

#### 3.4.1. Parameter Tuning

**Figure 13:**
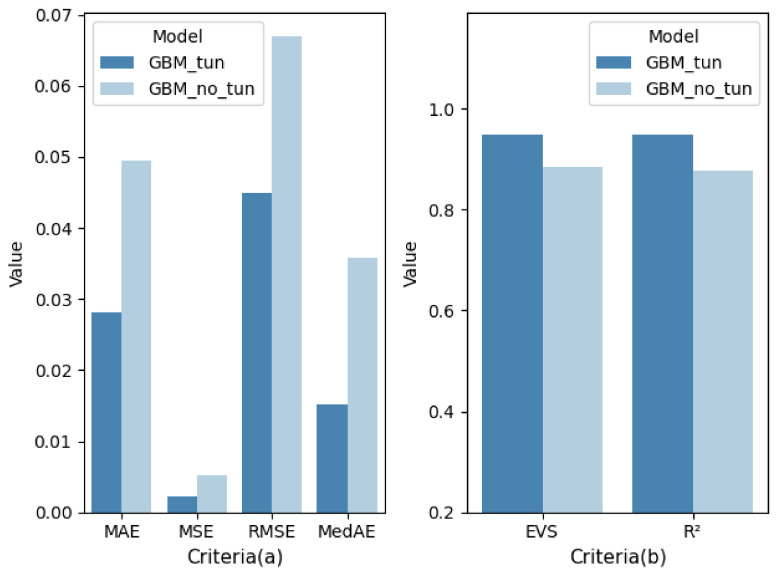
Comparison before and after LightGBM tuning by 10-fold cross-validation under different evaluation criteria: (**a**) The lower the criteria value, the better the model performance; (**b**) The higher the criteria value, the better the model performance.

After the grid search, the determined LightGBM parameters are shown in Table 3. Figure 13 shows the comparison of model performance before and after tuning. As shown in Figure 13, after parameter tuning, LightGBM obtained better performance and can more accurately predict the patient’s VCPLAT.

#### 3.4.2. Comparison with Four Machine Learning Models

**Figure 14:**
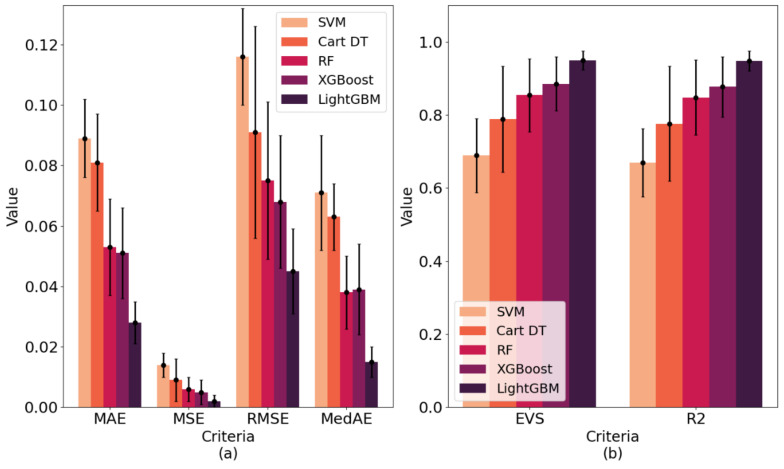
Prediction performances of five machine learning models by 10-fold cross-validation under different evaluation criteria: (**a**) The lower the criteria value, the better the model performance; (**b**) The higher the criteria value, the better the model performance. The data types presented in (**a**,**b**) are the results of the 10-fold cross-validation under each regression indicators in Table 4. The length of the histogram represents the mean of the cross-validation, and the error bars represent the standard deviation.

**Figure 15:**
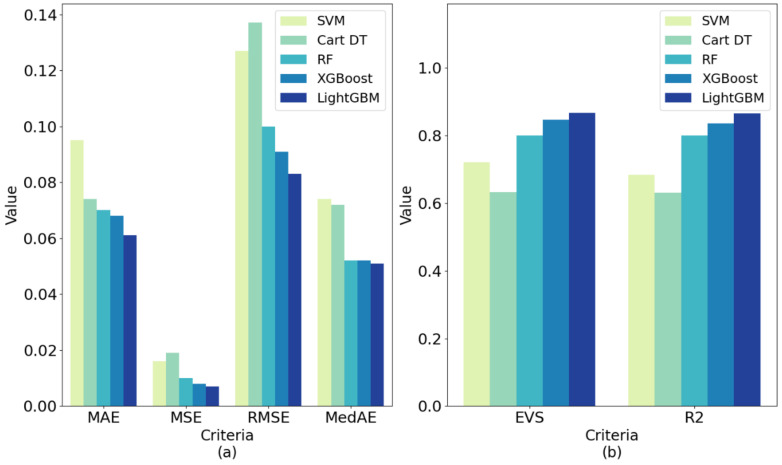
Prediction performances of five machine learning models on test set under different evaluation criteria: (**a**) The lower the criteria value, the better the model performance; (**b**) The higher the criteria value, the better the model performance. The data types presented in (**a**,**b**) are the results of the test set under each regression indicators in Table 5. The length of the histogram represents evaluation value, reflecting the difference between the predicted value and the true value.

In order to further verify the superiority of the proposed model, we compared the results of LightGBM with the following four commonly used machine learning models: support vector machine (SVM), cart decision tree (Cart DT), random forest (RF) and XGBoost. The optimal feature subsets selected by correlation analysis and the RFECV method were used for modeling. The comparison results of each model under different evaluation criteria are shown in Table 4, which are the average of the 10-fold cross-validation results (mean ± std). The comparison results on test set are shown in Table 5. Figure 14 (corresponding to Table 4) and Figure 15 (corresponding to Table 5) show more intuitively the performance scores of five models under six performance metrics.

We used the Friedman-Nemenyi statistical test to verify the significance of the 10-fold cross-validation results in Table 4. At the significance level α = 0.05, we used Friedman statistic *τ_F_* and the corresponding *p*-value to provide the quantitative information for the significance of the difference between methods.

**Figure 16:**
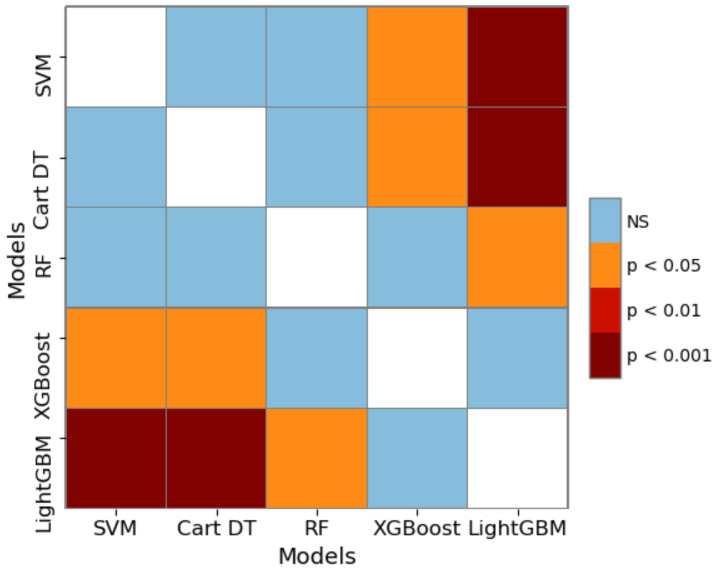
The *p*-value matrix heat map of Friedman test under criterion MAE. The data is from Table 7. NS stands for insignificant. The darker the color, the more significant the difference between the algorithms.

**Figure 17:**
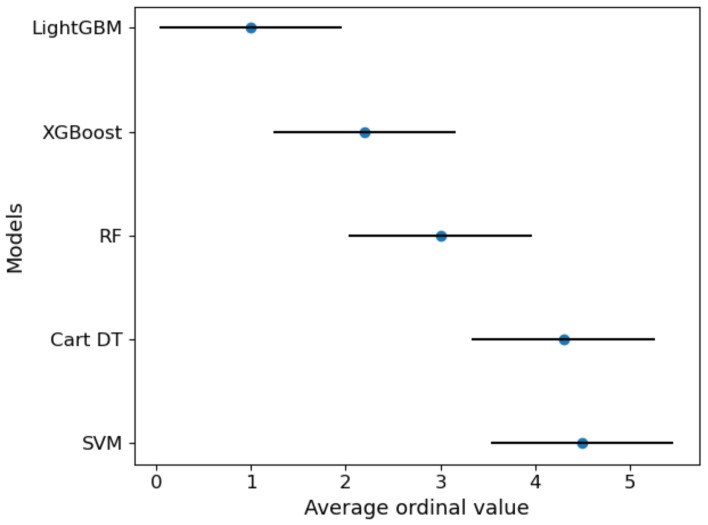
Friedman-Nemenyi test chart of 5 models under criterion MAE (CD = 1.928). The vertical axis in the figure shows each algorithm, and the horizontal axis is the average ordinal value. For each algorithm, a dot is used to display its average sequence value, and the horizontal line segment with the dot as the center represents the size of the critical value range. If the horizontal line segments of the algorithms overlap, it means that there is no significant difference between the two algorithms, otherwise, a significant difference exists.

Table 6 shows the calculation results of the Friedman Nemenyi statistical test, i.e., the average ordinal value and statistics of each algorithm in the 10-fold cross-validation under each criterion. Taking the criterion MAE as an example, the Friedman test *p*-value matrix of 5 algorithm is shown in Table 7 and Figure 16. In Figure 16, the blue represents no-significance (NS), and the darker the red, the more significant the difference in algorithm performance. Figure 17 visually showed the results after Nemenyi post-hoc test under criterion MAE.

## 4. Discussion

From the perspective of data mining and machine learning, we proposed a VCPLAT prediction model combining RFECV and LightGBM to help judge ventilatory pump failure. We target five pediatric neuromuscular diseases and analyzed 2518 lines of medical records of 704 patients with neuromuscular disorders from an American Clinic to verify the prediction model. Figure 8 shows that the overall distribution of VCPLAT in patients with SMA type 3 is relatively high, followed by DMD. The distribution of VCPLAT in patients with SMA type 1 is the lowest while it is more concentrated in CM patients.

As can be seen from Table 4 and Figure 14, the best performing model among all models was LightGBM. Its MAE, MSE, RMSE and MedAE in 10-fold cross-validation were 0.028, 0.002, 0.045 and 0.015, respectively, which are lower than the other machine learning models. Its EVS and R^2^ in 10-fold cross-validation were 0.949 and 0.948, respectively, which are higher than the other machine learning models. Therefore, the VCPLAT prediction model based on LightGBM achieved the best results. Table 5 and Figure 15 show that the LightGBM generated after training also achieved good performance on the test set. Its EVS, MAE, MSE, RMSE, R^2^ were superior to other methods with 0.867, 0.061, 0.007, 0.083, 0.051 and 0.865, respectively. This shows that LightGBM has good generalization performance and can be applied to VCPALT prediction of new samples.

Friedman-Nemenyi statistical test confirms the significance of the research results. Table 6 confirms that no matter which evaluation standard is used, the performance of the algorithms is significantly different (*τ_F_* > 2.634). Table 7, Figure 16 and Figure 17 further distinguish the significance between the algorithms. The results show that the boosting ensemble learning algorithms (such as XGBoost or LightGBM) is significantly better than Cart DT, SVM and other methods within 95% confidence interval. LightGBM is better than XGBoost, but the difference is not very significant.

From the results, correlation analysis and feature selection indicate that AGE, VCSIT, LASTVC, MIC, PCF, ETCO2MAX, BICEPSR, WRISTR and APCF have a greater influence on the prediction of the VCPLAT. Therefore, doctors need to focus on these indicators for pediatric patients with Duchenne muscular dystrophy, congenital myopathy and spinal muscular atrophy type 1–3.

After the VCPLAT prediction model was established, once the predicted value of the patient’s VCPLAT was obtained, the doctor can take targeted measures according to the value of the VCPLAT.

Compared with similar works in medical science, we provided a complete system of VCPLAT prediction methods and implementation process from data mining, bringing method innovation to the analysis in the medical field. Existing studies have established disease severity judgment and treatment methods based on VCPLAT but have not yet solved the key VCPLAT prediction problem. This study is based on a data-driven approach to fill this gap, which is helpful to assist doctors in subsequent diagnosis and treatment. At present, medical staff mainly rely on real-time VC monitoring to formulate treatment plans. Vital capacity changes are related to the patient, and it is difficult to determine VCPLAT, which leads to a delay in the treatment plan. The methods proposed in this paper can predict the patient’s VCPLAT in advance, and doctors can take timely countermeasures, thereby effectively improving the patient’s condition and reducing the probability of ventilatory pump failure.

Compared with similar works in machine learning, we have verified through experiments that machine learning regression methods can achieve good results in the prediction of important medical indicators. Among them, ensemble boosting learning methods have significant advantages. Existing research generally focused on the diagnosis and classification of neuromuscular diseases and pay less attention to the analysis and prediction of important indicators of patients. The methods proposed in this article realizes the innovation of machine learning in the medical application of neuromuscular diseases.

## 5. Conclusions

Comparing and analyzing the experimental results, we can draw the following conclusions: (1) Correlation Analysis and RFECV can screen valuable parts from many medical indicators and provide high-quality input variables for prediction model. (2) The prediction model combining RFECV and LightGBM algorithm performed well in both cross validation and test set evaluation, which can accurately and effectively predict VCPLAT values in patients with neuromuscular disorders.

The model proposed in this paper can provide guidance and aid decision-making for doctors in clinical diagnosis and treatment and has the significance of theoretical research and practical application. First, the model greatly reduces the number of medical indicators needed to determine the value of VCPLAT. This can reduce the number of testing items for patients.

Second, the model can accurately predict the patient’s VCPLAT. The VCPLAT is an indication of disease severity for pediatric diseases. For Duchenne muscular dystrophy, the progression rate of disease is related to VCPLAT. Doctors can use the predictive value of VCPLAT to help predict the severity of ventilatory pump failure for patients with Duchenne muscular dystrophy. So, doctors can prepare in advance and take corresponding protective measures such has indicating when to introduce lung volume recruitment to possibly preserve vital capacity, which enables patients to be treated promptly and properly when ventilatory pump failure occurs.

Third, the model can indicate which medical indicators have an impact on the VCPLAT. The model provides guidance for medical research. Experts and scholars in the medical field can analyze the correlation between various indicators and the VCPLAT from a pathological perspective.

## 6. Limitations and Further Study

The study in this paper has the following limitations for further research.

First, due to the difficulty in collecting medical data and the rarity of the disease, the amount of data used in this research is still small. With more samples accumulated and collected in the future, the model can be further optimized and adjusted.

Second, as a prediction target, VCPLAT is applicable to a small range of diseases. VCPLAT occurs in normal people at the age of 20, while it occurs earlier in children with Motor neuron disease (NMD). Therefore, the entire data concerning VCPLAT can only be for cases of pediatric onset diseases, such as Duchenne, SMA 1–3, and congenital myopathies. This study will choose more widely applicable indicators as prediction targets, such as End-Tidal Carbon Dioxide (ETCO2) and Pulse Oximetry (SPO2).

Third, this study temporarily did not consider the impact of patients’ disease progression and treatment methods on ventilatory pump failure due to lack of enough relevant data, but only predicted VCPLAT from the perspective of data mining. Each disease has a different rate of progression from the VCPLAT and actual respiratory failure is more dependent on how the doctors treat the patients (with CNVS and MIE or Cough Assist) than on how weak the patient is. Therefore, the model proposed in this study is more suitable for patients with Duchenne muscular dystrophy since evolution rates are related to the VCPLAT for this condition. As for other diseases, the predictive nature for future complications depends on other factors such as rate of progression and individual clinical care. In future study, we will further consider the effects of treatment methods and other factors on ventilatory pump failure.

## Figures and Tables

**Table 1:** Description of partial medical indicators.

No.	Name	Description	Type	Value Range	Mean Value	Std.
1	HE_SHE	Gender	Categorical	Male or Female	/	/
2	BICEPSL	Left bicep strength	Categorical	(0 to 5+)	/	/
3	AGE	Age	Numeric	(0, 83)	15.65	12.5
4	VCSIT	Sitting vital capacity	Numeric	(0, 6060)	887.3	921
5	VCSUPINE	Supine vital capacity	Numeric	(0, 5300)	133.3	483.5
6	MIC	Maximum blowing capacity	Numeric	(0, 5980)	621	872.7
7	PCF	Peak cough flow rate	Numeric	(0, 12.5)	1.58	1.99
8	SAO2LOW	Observed low oxygen content	Numeric	(0, 99)	73.17	41.5
9	SAO2HIGH	Observed high oxygen content	Numeric	(0, 99)	73.94	41.9
10	ETCO2MAX	Maximum end-tidal carbon dioxide	Numeric	(0, 72)	27.66	17.88
11	ROMLTKNEE	Range of motion of left knee	Numeric	(0, 99)	4.432	13.4
12	ROMHIPEXL	Range of motion of left hip extension	Numeric	(0, 99)	4.141	9.07
13	VCPLAT	Vital capacity plateau value	Numeric	(0, 3350)	369.8	810.2
14	AGEPLAT	Lifetime maximum age	Numeric	(0, 17)	0.603	2.61

**Table 2:** Muscle strength feature conversion.

Original Value	0	1−	1	1+	2−	2	2+	3−	3	3+	4−	4	4+	5−	5	5+
Converted Value	1	2	3	4	5	6	7	8	9	10	11	12	13	14	15	16

Muscle strength is mainly divided into six levels of 0–5, of which, 0 is completely paralyzed, 1 is the slight contraction of the muscle and the joint cannot move, 2 is full range of joint movements under weight loss, 3 means muscle can resist gravity for full range of joint movement but not resistance, 4 means muscle can exercise against gravity and resistance, and 5 is normal. The severity increases sequentially from 5 to 0, of which, each value additionally has a + and − value surrounding it, i.e., 2− is between 2 and 1+, and 2+ is between 2 and 3−.

**Table 3:** LightGBM parameters.

Parameter	Explanation	Value
n_estimators	Number of iterations	700
max_depth	The maximum depth of the tree	5
num_leaves	The number of leaf nodes	12
max_bin	An integer representing the maximum number of buckets.	15
min_child_weight	The sum of the smallest hessian on a leaf node.	0.001
lambda_l1	Regularization parameters that can reduce overfitting.	0.001
lambda_l2	Regularization parameters that can reduce overfitting.	0
min_split_gain	Minimum gain to perform segmentation	0
learning_rate	Smaller learning rate can improve accuracy.	0.085
min_data_per_leaf	The minimum number of samples per leaf node.	1
sub_row	Sample sampling can make bagging run faster and prevent overfitting.	0.6
bagging_freq	The frequency of bagging.	0
sub_feature	Feature sub-sampling can be used to prevent overfitting and improve training speed.	0.8

**Table 4:** Comparison of the machine learning model results.

	Criteria	EVS	MAE	MSE	RMSE	MedAE	R^2^
Model							
SVM		0.689 ± 0.101	0.089 ± 0.013	0.014 ± 0.004	0.116 ± 0.016	0.071 ± 0.019	0.669 ± 0.093
Cart DT		0.788 ± 0.145	0.081 ± 0.016	0.009 ± 0.007	0.091 ± 0.035	0.063 ± 0.011	0.776 ± 0.157
RF		0.854 ± 0.100	0.053 ± 0.016	0.006 ± 0.004	0.075 ± 0.026	0.038 ± 0.012	0.848 ± 0.103
XGBoost		0.885 ± 0.074	0.051 ± 0.015	0.005 ± 0.004	0.068 ± 0.022	0.039 ± 0.015	0.877 ± 0.083
LightGBM		0.949 ± 0.026	0.028 ± 0.007	0.002 ± 0.002	0.045 ± 0.014	0.015 ± 0.005	0.948 ± 0.027

Data presented in the table are the results of 10-fold cross-validation under different evaluation criteria (mean ± std).

**Table 5:** Test set evaluation results of different machine learning models.

	Criteria	EVS	MAE	MSE	RMSE	MedAE	R^2^
Model							
SVM		0.721	0.095	0.016	0.127	0.074	0.683
Cart DT		0.633	0.074	0.019	0.137	0.072	0.631
RF		0.801	0.070	0.010	0.100	0.052	0.801
XGBoost		0.847	0.068	0.008	0.091	0.052	0.836
LightGBM		0.867	0.061	0.007	0.083	0.051	0.865

Data presented in the table are the results of test set evaluation under different criteria. The value reflects the difference between the predicted value and the true value.

**Table 6:** The average ordinal value of each algorithm and Friedman statistics.

	Model	SVM	Cart DT	RF	XGBoost	LightGBM	*τ_F_*
Criteria							
EVS		4.6	4.4	2.9	1.9	1.2	79.24
MAE		4.5	4.3	3	2.2	1	54.38
MSE		4.6	4.4	2.9	1.9	1.2	79.24
RMSE		4.6	4.4	2.9	1.9	1.2	79.24
MedAE		4.4	4.4	2.5	2.6	1.1	34.69
R^2^		4.6	4.4	2.9	1.9	1.2	79.24

The data type is the average ordinal value of each algorithm on 10 sets of data (derived from ten-fold cross-validation) under 6 criteria.

**Table 7:** The Friedman test *p*-value matrix under criterion MAE.

	Model	SVM	Cart DT	RF	XGBoost	LightGBM
Criteria						
SVM		\	0.9	0.210897	0.010068	0.001
Cart DT		0.9	\	0.351727	0.024841	0.001
RF		0.210897	0.351727	\	0.763265	0.037722
XGBoost		0.010068	0.024841	0.763265	\	0.437421
LightGBM		0.001	0.001	0.037722	0.437421	\

The data type is the *p*-value of Friedman test.

## Data Availability

The data presented in this study are available on request from the corresponding author with required ethical review. The data are not publicly available due to patient privacy protection.

## Appendix A

Table A1 is a list of all the 40 medical features that are considered in this study.

**Table A1:** 40 medical features and their explanations.

No.	Abbreviations	Explanation	Type
1	NAME	name of patients	Categorical
2	HE_SHE	gender	Categorical
3	AGE	age	Numeric
4	FALLPERMO	fall to the ground every month	Numeric
5	SHOULDERL	left shoulder flexion and extension strength	Categorical
6	SHOULDERR	right shoulder flexion and extension strength	Categorical
7	BICEPSL	left bicep strength	Categorical
8	BICEPSR	right bicep strength	Categorical
9	WRISTL	left wrist extension	Categorical
10	WRISTR	right wrist extension	Categorical
11	MPHIPFLLT	left hip flexion strength	Categorical
12	MPHIPFLRT	right hip flexion strength	Categorical
13	MPHIPEXLT	left hip extension strength	Categorical
14	MPHIPEXRT	right hip extension strength	Categorical
15	MPQUADLT	left quadriceps strength	Categorical
16	MPQUADRT	right quadriceps strength	Categorical
17	MPDORSILT	left dorsiflexion strength	Categorical
18	MPDORSIRT	right dorsiflexion strength	Categorical
19	WALK10YDS	how fast the patient can walk within 10 yards	Numeric
20	UPFROMSIT	time for patients to stand up from sitting on the floor	Numeric
21	VCSIT	sitting vital capacity	Numeric
22	VCSUPINE	supine vital capacity	Numeric
23	MIC	maximum blowing capacity (maximum air stack volume)	Numeric
24	PCF	peak cough flow rate	Numeric
25	PCFMIC	peak cough flow measured from MIC	Numeric
26	APCF	assisted cough peak flow	Numeric
27	SAO2LOW	observed low oxygen content	Numeric
28	SAO2HIGH	observed high oxygen content	Numeric
29	ETCO2MAX	maximum end-tidal carbon dioxide	Numeric
30	LASTVC	sitting vital capacity at the last visit	Numeric
31	ROMDORSIL	range of motion of left ankle dorsiflexion	Numeric
32	ROMDORSIR	range of motion of right ankle dorsiflexion	Numeric
33	ROMLTKNEE	range of motion of left knee	Numeric
34	ROMRTKNEE	range of motion of right knee	Numeric
35	ROMHIPEXL	range of motion of left hip extension	Numeric
36	ROMHIPEXR	range of motion of right hip extension	Numeric
37	ROMITBLT	range of motion of left iliotibial bandage	Numeric
38	ROMITBRT	range of motion of right iliotibial bandage	Numeric
39	VCPLAT	vital capacity plateau value	Numeric
40	AGEPLAT	lifetime maximum age	Numeric

## References

[1] Pandit C., Waters K., Jones K., Young H., Fitzgerald D. (2015). Can daytime measures of lung function predict respiratory failure in children with neuromuscular disease?. Paediatr. Respir. Rev..

[2] Rideau Y., Jankowski L., Grellet J.J.M. (1981). Respiratory function in the muscular dystrophies. Muscle Nerve: Off. J. Am. Assoc. Electrodiagn. Med..

[3] Bach J.R., Tuccio M.C., Khan U., Saporito L.R. (2012). Vital capacity in spinal muscular atrophy. Am. J. Phys. Med. Rehabil..

[4] Chiou M., Bach J.R., Jethani L., Gallagher M.F. (2017). Active lung volume recruitment to preserve vital capacity in Duchenne muscular dystrophy. J. Rehabil. Med..

[5] Ye Y., Liu C., Zemiti N., Yang C. Optimal Feature Selection for EMG-Based Finger Force Estimation Using LightGBM Model. Proceedings of the 2019 28th IEEE International Conference on Robot and Human Interactive Communication (RO-MAN).

[6] Bolourani S., Brenner M., Wang P., McGinn T., Hirsch J.S., Barnaby D., Zanos T.P. (2021). A machine learning prediction model of respiratory failure within 48 h of patient admission for COVID-19: Model development and validation. J. Med. Internet Res..

[7] Bolourani S., Wang P., Patel V.M., Manetta F., Lee P.C. (2020). Predicting respiratory failure after pulmonary lobectomy using machine learning techniques. Surgery.

[8] Burdick H., Lam C., Mataraso S., Siefkas A., Braden G., Dellinger R.P., McCoy A., Vincent J.-L., Green-Saxena A. (2020). Prediction of respiratory decompensation in Covid-19 patients using machine learning: The READY trial. Comput. Biol. Med..

[9] Jia Y., Kaul C., Lawton T., Murray-Smith R., Habli I. (2021). Prediction of weaning from mechanical ventilation using convolutional neural networks. Artif. Intell. Med..

[10] Ramli A.A., Zhang H., Hou J., Liu R., Liu X., Nicorici A., Aranki D., Owens C., Prasad P., McDonald C. (2021). Gait Characterization in Duchenne Muscular Dystrophy (DMD) Using a Single-Sensor Accelerometer: Classical Machine Learning and Deep Learning Approaches. arXiv.

[11] Greco A., Chiesa M.R., Da Prato I., Romanelli A.M., Dolciotti C., Cavallini G., Masciandaro S.M., Scilingo E.P., Del Carratore R., Bongioanni P. (2021). Using blood data for the differential diagnosis and prognosis of motor neuron diseases: A new dataset for machine learning applications. Sci. Rep..

[12] Pattichis C.S., Schizas C.N. (1996). Genetics-based machine learning for the assessment of certain neuromuscular disorders. IEEE Trans. Neural Netw..

[13] Valero-Cuevas F.J., Hoffmann H., Kurse M.U., Kutch J.J., Theodorou E.A. (2009). Computational models for neuromuscular function. IEEE Rev. Biomed. Eng..

[14] Yousefi J., Hamilton-Wright A. (2014). Characterizing EMG data using machine-learning tools. Comput. Biol. Med..

[15] Subasi A. (2013). Classification of EMG signals using PSO optimized SVM for diagnosis of neuromuscular disorders. Comput. Biol. Med..

[16] Subasi A. (2012). Medical decision support system for diagnosis of neuromuscular disorders using DWT and fuzzy support vector machines. Comput. Biol. Med..

[17] Güler N.F., Koçer S. (2005). Use of support vector machines and neural network in diagnosis of neuromuscular disorders. J. Med. Syst..

[18] Srivastava T., Darras B.T., Wu J.S., Rutkove S.B. (2012). Machine learning algorithms to classify spinal muscular atrophy subtypes. Neurology.

[19] Choi S.B., Park J.S., Chung J.W., Yoo T.K., Kim D.W. Multicategory classification of 11 neuromuscular diseases based on microarray data using support vector machine. Proceedings of the 2014 36th Annual International Conference of the IEEE Engineering in Medicine and Biology Society.

[20] Al Shalabi L., Najjar M., Al Kayed A. (2006). A framework to deal with missing data in data sets. J. Comput. Sci..

[21] Minakshi, Vohra R., Gimpy (2014). Missing value imputation in multi attribute data set. Int. J. Comput. Sci. Inf. Technol..

[22] Malarvizhi R., Thanamani A.S. (2012). K-nearest neighbor in missing data imputation. Int. J. Eng. Res. Dev..

[23] Pujianto U., Wibawa A.P., Akbar M.I. K-Nearest Neighbor (K-NN) based Missing Data Imputation. Proceedings of the 2019 5th International Conference on Science in Information Technology (ICSITech).

[24] Sallaby A.F., Azlan A. (2021). Analysis of Missing Value Imputation Application with K-Nearest Neighbor (K-NN) Algorithm in Dataset. IJICS Int. J. Inform. Comput. Sci..

[25] Albon C. (2018). Machine Learning with Python Cookbook: Practical Solutions from Preprocessing to Deep Learning.

[26] Anusuya V., Gomathi V. (2021). An Efficient Technique for Disease Prediction by Using Enhanced Machine Learning Algorithms for Categorical Medical Dataset. Inf. Technol. Control.

[27] Cui L., Xie X., Shen Z. (2018). Prediction task guided representation learning of medical codes in EHR. J. Biomed. Inform..

[28] Gogtay N.J., Thatte U.M. (2017). Principles of correlation analysis. J. Assoc. Physicians India.

[29] Jung I.-Y., Chae J.H., Park S.K., Kim J.H., Kim J.Y., Kim S.J., Bang M.S. (2012). The correlation analysis of functional factors and age with duchenne muscular dystrophy. Ann. Rehabil. Med..

[30] El Aboudi N., Benhlima L. Review on wrapper feature selection approaches. Proceedings of the 2016 International Conference on Engineering & MIS (ICEMIS).

[31] Wang C., Xiao Z., Wu J. (2019). Functional connectivity-based classification of autism and control using SVM-RFECV on rs-fMRI data. Phys. Med..

[32] Rashid M., Singh H., Goyal V., Parah S.A., Wani A.R. (2021). Big data based hybrid machine learning model for improving performance of medical Internet of Things data in healthcare systems. Healthcare Paradigms in the Internet of Things Ecosystem.

[33] Akyol K., Atila Ü. (2019). A study on performance improvement of heart disease prediction by attribute selection methods. Acad. Platf. J. Eng. Sci..

[34] Ke G., Meng Q., Finley T., Wang T., Chen W., Ma W., Ye Q., Liu T.-Y. (2017). Lightgbm: A highly efficient gradient boosting decision tree. In Proceedings of the Advances in Neural Information Processing Systems. Adv. Neural Inf. Process. Syst..

[35] Bentéjac C., Csörgő A., Martínez-Muñoz G. (2021). A comparative analysis of gradient boosting algorithms. Artif. Intell. Rev..

[36] Bahad P., Saxena P. Study of adaboost and gradient boosting algorithms for predictive analytics. Proceedings of the International Conference on Intelligent Computing and Smart Communication 2019.

[37] Chen C., Zhang Q., Ma Q., Yu B. (2019). LightGBM-PPI: Predicting protein-protein interactions through LightGBM with multi-information fusion. Chemom. Intell. Lab. Syst..

[38] Wang D., Zhang Y., Zhao Y. LightGBM: An effective miRNA classification method in breast cancer patients. Proceedings of the 2017 International Conference on Computational Biology and Bioinformatics.

[39] Amirgaliyev Y., Shamiluulu S., Merembayev T., Yedilkhan D. Using Machine Learning Algorithm for Diagnosis of Stomach Disorders. Proceedings of the International Conference on Mathematical Optimization Theory and Operations Research.

